# Effect of preceding exercise on cerebral and splanchnic vascular responses to mental task

**DOI:** 10.1186/1880-6805-31-17

**Published:** 2012-06-25

**Authors:** Nami Someya, Tsukasa Ikemura, Naoyuki Hayashi

**Affiliations:** 1 Graduate School of Life Science and Systems Engineering, Kyushu Institute of Technology, Kitakyushu, Fukuoka, 808-0196, Japan; 2 Graduate School of Human-Environment Studies, Kyushu University, Kasuga, Fukuoka, 816-8580, Japan; 3 Institute of Health Science, Kyushu University, Kasuga, Fukuoka, 816-8580, Japan

**Keywords:** Mental stress, Acute exercise, Superior mesenteric artery, Middle cerebral artery

## Abstract

**Background:**

To investigate the effect of preceding acute exercise on the peripheral vascular response to a mental task, we measured splanchnic and cerebral blood flow responses to performing a mental task after exercise and resting.

**Methods:**

In the exercise trial, 11 males exercised for 30 min on a cycle ergometer with a workload set at 70% of the age-predicted maximal heart rate for each individual. After a 15-min recovery period, the subjects rested for 5 min for pre-task baseline measurement and then performed mental arithmetic for 5 min followed by 5 min of post-task measurement. In the resting trial, they rested for 45 min and pre-task baseline data was obtained for 5 min. Then mental arithmetic was performed for 5 min followed by post-task measurement. We measured the mean blood velocity in the middle cerebral artery and superior mesenteric artery and the mean arterial pressure.

**Results:**

Mean arterial pressure and mean blood velocity in the middle cerebral artery were significantly higher than the baseline during mental arithmetic in both exercise and resting trials. Mean blood velocity in the middle cerebral artery during mental arithmetic was greater in the control trial than the exercise trial. Mean blood velocity in the superior mesenteric artery showed no significant change during mental arithmetic from baseline in both trials.

**Conclusion:**

These results suggest that acute exercise can moderate the increase in cerebral blood flow induced by a mental task.

## Background

Understanding physiological responses to mental tasks is useful for the establishment of stress management. Although there is a general consensus that regular exercise exerts beneficial effects on cardiovascular fitness [1], the effect of acute exercise on cardiovascular response to mental tasks is controversial. According to a systematic review [2], 10 studies reported that acute exercise attenuated the pressor response to mental tasks, but five did not. In addition, the mechanism of exercise-induced acute attenuation of pressor response, if any, is not established.

A previous study reported that the total peripheral resistance is lower during post-exercise resting and mental task compared to the corresponding period of the non-exercise trial [3]. In addition, Brownley et al. [4] reported that the responses of plasma norepinephrine and epinephrine during post-exercise mental tasks were reduced with concomitant attenuation of pressor response. Based on these results, reduction of peripheral vascular tone due to alteration of sympathetic control is likely to be one of the plausible mechanisms for the post-exercise attenuation of pressor responses to mental tasks. Nevertheless, the region responsible for the reduction of total vascular resistance is unknown. Mental tasks exert differential vascular responses among various vasculatures; for example, they induce vasodilation in the cerebral region [5-7] and vasoconstriction in the splanchnic region [8-12]. Thus, the increase of vasodilation and/or the decrease of vasoconstriction in the responsible regions could contribute to the reduction of total peripheral resistance, and consequently to the attenuation of pressor responses to mental tasks.

Cerebral blood flow is coupled with brain metabolism [13], and sympathetic activation seems to be less effective for the control of cerebral blood flow, except under extreme conditions [14]. Thus, it is not feasible for lower sympathetic activation to mental tasks in the cerebral region to be associated with the post-exercise attenuation of pressor responses to mental tasks. On the other hand, the splanchnic region is one of the main regions where vasoconstriction occurs during mental tasks, increasing total peripheral resistance. Splanchnic vasoconstriction was, at least partly, induced by sympathetic nervous activity [9]. Thus, it is plausible that decreased vasoconstriction in the splanchnic region, which is possibly induced by lower sympathetic activation, could be associated with post-exercise attenuation of the pressor response to mental tasks.

In this context, we hypothesized that post-exercise attenuation of the pressor response to mental tasks is associated with decreased vasoconstriction in the splanchnic region, but not with increased vasodilation in the cerebral region. To test this hypothesis, we measured the blood velocity as index of blood flow responses in the middle cerebral artery (MCA) and superior mesenteric artery (SMA) during mental tasks after exercise.

## Methods

### Subjects

Eleven healthy, untrained males who were 25 ± 5 years old (mean ± SD) and 170 ± 6 cm tall and weighed 63 ± 10 kg participated in the study. The subjects were normotensive, non-obese, not taking any medication, and had no history of autonomic dysfunction or cardiovascular disease. The Ethics Committee of the Institute of Health Science, Kyushu University, approved the experimental protocols, and all subjects provided written informed consent to participate. All protocols conformed to the Declaration of Helsinki.

### Protocols

The subjects arrived at the laboratory at 0800 to 1000 after having abstained from caffeine, intensive exercise, and smoking for 12 h. Each subject ate the control meal (7.6 g protein, 4.4 g fat and 32.5 g carbohydrate with a total caloric value of 200 kcal; Calorie Mate Jelly; Otsuka Pharmaceutical, Tokyo, Japan) at least 1 h before arriving. The arrival time each day was almost the same within subjects. To acclimate to the experimental environment, subjects rested for at least 30 min before the measurement. The protocol was conducted in a quiet room with the subject in a semi-recumbent position with the hips extended to approximately 130°.

**Figure 1:**
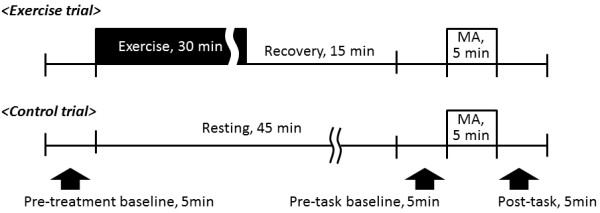
**Protocol of the present study.** In the exercise trial, the subjects exercised for 30 min after 5 min of pre-treatment baseline. Fifteen minutes after the end of exercise, the subjects rested for 5 min for pre-task baseline measurement and then performed mental arithmetic (MA) for 5 min followed by 5 min of post-task measurement. In the control trial, the same subjects performed MA almost same time-sequence with exercise trial except for exercise.

In the exercise trial, the subjects exercised for 30 min on a cycle ergometer (232c XL, Combi, Japan) with a workload set at 70% of the age-predicted maximal heart rate (HR) of each individual after 5 min of pre-treatment baseline measurements (Figure 1). At the cessation of exercise, each subject was asked to report his rate of perceived exertion, and rested for 15 min as a recovery phase. Then, after the pre-task baseline data was obtained for 5 min, subjects performed mental arithmetic (MA) as a mental task for 5 min. The subjects were requested to subtract 7 or 13 from 500 or 1,000 and to answer verbally as fast as possible. In the control trial, the same subjects performed MA almost same time sequence with exercise trial except for exercise; after 5 min of pre-treatment baseline, the subject rested for 45 min (same time sequence with 30-min exercise and 15-min recovery phase). Then pre-task baseline data was obtained for 5 min followed by MA for 5 min. In both trials, within 1 min after finishing the MA, each subject was asked to report his rate of the perceived stress using a visual analogue scale, and then rested for 5 min for the post-task measurements. Each trial was performed on a separate day in randomized order.

### Measurements

During the pre-treatment baseline, pre-task baseline, MA, and post-task period, the HR, mean arterial pressure (MAP), systolic blood pressure (SBP), diastolic blood pressure (DBP), and mean blood velocities (MBVs) in both MCA and SMA were recorded. HR was recorded continuously using an electrocardiograph (MEG2100; Nihon-Kohden, Tokyo, Japan). Beat-by-beat MAP, SBP, and DBP were monitored using Finometer on the left middle finger (Finapres Medical System, Amsterdam, the Netherlands). MBV of the right MCA was obtained by transcranial Doppler ultrasonography (WAKI; Atys Medical, St Genislaval, France). A 2 MHz Doppler probe was placed at the right temporal window and fixed with an adjustable headband. The velocity waveform of the MCA was obtained 47 to 51 mm from the skin surface. Simultaneous pulsed and echo Doppler ultrasound flowmetry was used to measure MBV in the SMA, as described previously [15,16]. In brief, we used a curved-array Doppler-scan probe operating at a pulsed Doppler frequency of 3.3 MHz (LOGIQ3; GE Medical Systems, Salem, CT, USA). The Doppler-beam insonation angle was maintained at no more than 60° relative to the blood vessel. All physiological signals were sampled at 20 kHz using an A/D converter (PowerLab 8 s; ADInstruments, Colorado Springs, CO, USA). The spectra of the signals related to antegrade and retrograde velocities in the SMA and the electrocardiography were analyzed using our own software, and beat-by-beat MBV in the SMA values were calculated. During exercise, electrocardiogram was continuously recorded and HR was monitored spontaneously (OEC-6201, Nihon-Kohden, Japan). Rate of the perceived stress was assessed using a visual analog scale, marking on a 100-mm line the extent to which they had experienced stress during the MA, with no stress and very stressful indicated at 0 and 100 mm, respectively.

### Data analysis

Minute-by-minute HR, MAP, SBP, and DBP values were calculated from the electrocardiogram and blood-pressure recordings. To obtain minute-by-minute MBV in the MCA, beat-by-beat MBV was averaged every minute. To obtain minute-by-minute MBV in the SMA data, the 10 largest values of the beat-by-beat MBV were averaged every minute, since lower values are mainly artifacts caused by respiration. We have previously obtained reliable velocity data using this method [15].

MBVs in the MCA and SMA were divided by MAP on a minute-by-minute basis to evaluate vascular conductance index (VCI). Minute-by-minute data for each variable were averaged over 5 min of pre-treatment baseline, pre-task baseline, and post-task period.

In four of eleven subjects, we successfully obtained high-quality Doppler recordings of the MCA and SMA at the same time. In another subject, we could not obtain sufficient Doppler recording from MCA, mainly due to the location of artery. Then we obtained the MBV data solely from the SMA in this subject. In three subjects, we could not obtain sufficient Doppler recordings from SMA, mainly due to the location of artery. Then we obtained the MBV data solely from the MCA in these subjects. In the remaining three subjects, we could not obtain sufficient Doppler recordings in the SMA due to abdominal gas; thus, we re-measured MBV in the SMA on other days. In these subjects, MBV data of MCA and SMA were obtained on separate days for the exercise and control trials. We included MBV data from the MCA in 10 subjects and from the SMA in eight subjects. Minute-by-minute data of HR and MAP from subjects who performed each trial twice were averaged in repeated measurements.

### Statistical analysis

Data are expressed as the mean ± SE. The difference in the pre-treatment baseline between exercise and control trials was evaluated using the paired *t*-test. The difference between the pre-treatment and pre-task baseline was also evaluated using the paired *t*-test for each trial. The paired *t*-test was also used to compare the rate of perceived stress between trials.

To evaluate the effect of exercise on the responses to MA, the main effect of time and trial, and their interactions were examined by two-way repeated ANOVA. When a significant *F* value was detected, this was examined further by Dunnett’s post-hoc test to assess the effect of time and the paired *t*-test to compare the values between trials at each time point.

Statistical significance was accepted at *P* <0.05. Statistical analyses were performed with SAS (ver. 8.2; SAS Institute, Cary, NC, USA) at the Computing and Communications Center, Kyushu University.

## Results

During the last 5 min of exercise session, the HR was 138 ± 2 bpm, corresponding to 71 ± 1% (68-73%) of age-predicted maximum HR or 57 ± 1% of HR reserve. The workload at the end of exercise was 104 ± 5 W and the rate of perceived exertion was 13.9 ± 0.4, corresponding to ‘somewhat hard’ to ‘hard’. There was no significant difference in the rates of perceived stress between exercise (58 ± 5) and control (64 ± 6) trials.

### Systemic circulation

**Figure 2:**
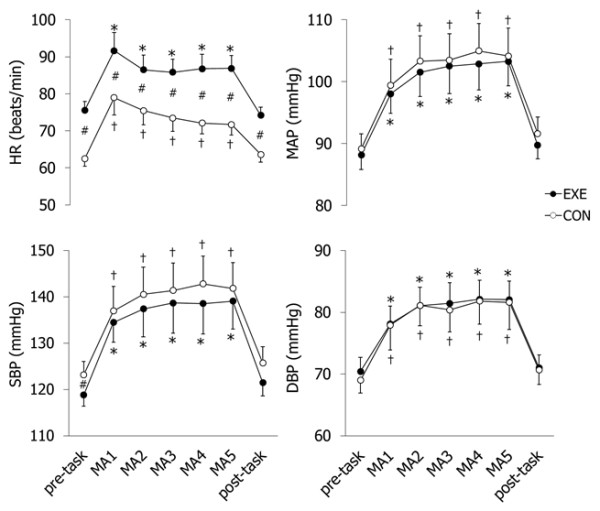
**Systemic circulatory responses to mental arithmetic (MA) during EXE (●) and CON (○) trials.** MA1, MA2, MA3, MA4, and MA5 show 1st, 2nd, 3rd, 4th, and 5th minute of MA. MA significantly increased HR, MAP, SBP, and DBP. *Difference from pre-task in EXE. †Difference from pre-task in CON. #Difference between EXE and CON trials (*P* <0.05).

The pre-treatment baseline of the HR, MAP, SBP, and DBP did not differ significantly between exercise and control trials (Table 1). In the exercise trial, the HR significantly increased, while SBP significantly decreased from pre-treatment to the pre-task baseline. MAP and DBP did not change significantly from pre-treatment to the pre-task baseline in both trials. HR, MAP, SBP, and DBP increased during MA from the pre-task baseline in both trials (Figure 2). HR was greater in the exercise trial than in the control trial throughout the MA and pre- and post-task periods. There were no significant differences between trials in MAP, SBP, and DBP.

**Table 1:** Pre-treatment and pre-task baseline values

	**EXE**		**CON**	
	**Pre-treatment**	**Pre-task**	**Pre-treatment**	**Pre-task**
HR (bpm)	61 ± 2	75 ± 2^a,b^	63 ± 2	62 ± 2
MAP (mmHg)	90 ± 2	88 ± 2	88 ± 2	89 ± 2
SBP (mmHg)	124 ± 2	119 ± 2^a,b^	121 ± 2	123 ± 3
DBP (mmHg)	70 ± 2	70 ± 2	69 ± 2	70 ± 2
MBV in MCA (m/s)	0.64 ± 0.02	0.62 ± 0.03	0.65 ± 0.02	0.64 ± 0.02
MBV in SMA (m/s)	0.38 ± 0.02	0.36 ± 0.01	0.40 ± 0.02	0.35 ± 0.02^a^
VCI in MCA (mm/s/mmHg)	7.2 ± 0.3	7.2 ± 0.4	7.4 ± 0.4	7.2 ± 0.4
VCI in SMA (mm/s/mmHg)	4.3 ± 0.2	4.2 ± 0.1	4.7 ± 0.3	4.0 ± 0.2^a^

Values before exercise and resting (pre-treatment) and before mental arithmetic (pre-task) in exercise (EXE) and control (CON) trials.

^a^Difference between pre-treatment and pre-task.

^b^Difference between EXE and CON trials (*P* <0.05).

bpm, beats per minute; DBP, diastolic blood pressure; HR, heart rate; MAP, mean arterial pressure; MBV, mean blood velocity; MCA, middle cerebral artery; SBP, systolic blood pressure; SMA, superior mesenteric artery; VCI, vascular conductance index.

### Peripheral circulation

The pre-treatment baseline of MBV in the MCA and SMA did not significantly differ between trials (Table 1). MBV and VCI in the MCA did not change significantly from pre-treatment to the pre-task baseline in both exercise and control trials. MBV and VCI in the SMA significantly decreased from pre-treatment to the pre-task baseline in the control trial but not in the exercise trial.

**Figure 3:**
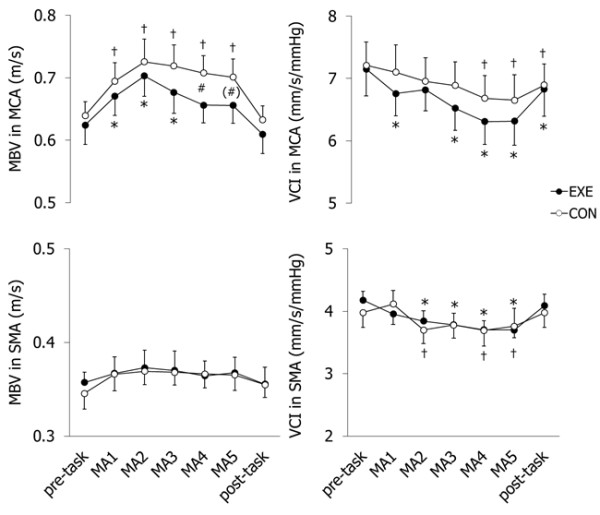
**Peripheral circulatory response to MA during EXE (●) and CON (○) trials.** MA increased in MBV in MCA and decreased in VCI in MCA and SMA. MBV in SMA did not change significantly. *Difference from pre-task in EXE. †Difference from pre-task in CON. #Difference between EXE and CON trials (P < 0.05). (#)Difference between EXE and CON trials (*P* = 0.09).

MBV in the MCA increased in the 1st to 3rd minute of MA in the exercise trial and throughout MA in the control trial (Figure 3). MBV in the MCA was significantly greater in the exercise trial than the control trial in the 4th minute of MA, and tended to be greater in the 5th minute of MA. MBV in the SMA did not change significantly in both trials. VCI in the MCA and SMA significantly decreased from the pre-task in both trials. There were no significant differences between trials in MBV in the SMA and VCI in the both arteries.

## Discussion

We investigated the effect of exercise on the peripheral vascular response to a mental task. We could not obtain post-exercise attenuation of the pressor response and splanchnic vasoconstriction with the mental task. Nevertheless, we found that preceding exercise attenuated the blood flow increase in the cerebral vasculature during the mental task, implying that acute exercise can modify the cerebral vascular response to mental tasks.

The mental task activated the systemic circulation in both exercise and control trials. In the present study, preceding exercise did not attenuate the pressor response to mental tasks. Hamer et al. [2] performed meta-analysis using 15 studies and suggested that significant attenuative effects of exercise on pressor response to mental tasks were observed when exercise intensity was greater than 60% of Vo_2max_ or 75% of age-predicted maximum HR. Nevertheless, significant effects were still observed at a lower intensity of exercise, such as 30 min at 50% of Vo_2max_[17], 20 min at 60% to 70% of the age-predicted maximum HR [3], and 20 min of 100 W cycling [18]. The exercise intensity in the present study was comparable to this range. Thus, lack of an attenuation effect of exercise on the pressor response cannot be necessarily attributed to lower exercise intensity. The review also implied that studies involving weaker stressors, weaker responders, and a longer period between exercise and mental tasks were least likely to show a significant effect [2]. Previous studies, showing a significant effect of exercise, reported that the mental task itself increased the HR by approximately 4 to 18 bpm [19,20] and MAP approximately by 8 to 17 mmHg [4,20] from baseline. In the present study, the increases in HR (approximately 12 bpm) and MAP (approximately 15 mmHg) during the mental task were within these ranges. In addition, the mental task was set at 20 min after the end of exercise in our protocol, shorter than the time at which consistent effects were observed in previous studies, that is, up to 30 min post-exercise [2]. Thus, we cannot determine the factor inducing no effect of exercise on pressor responses to mental tasks.

MBV and VCI in the MCA did not differ between pre-treatment and the pre-task baseline in both trials, suggesting that neither vasoconstriction nor vasodilation occurred in the cerebral region after the end of exercise, partly consistent with the previous study which reported no significant change in MCA during 1 to 8 min after the exercise at HR of 90, 120, and 150 bpm [21].

MBV in the MCA increased during the mental task in both trials. MBV in the MCA during the mental task was smaller in the exercise trial than the control trial, suggesting that preceding exercise suppresses the cerebral blood flow increase associated with the mental task. Cerebral blood flow is coupled with brain metabolism [13]. Thus the present result led us to hypothesize that blood flow increase was not enough to meet the increased metabolic demand and consequently attenuated performance of mental task. In turn, it was reported that mental task alter the blood flow distribution in the brain regions without significant change in global cerebral blood flow [22]. Based on this result, there is a possibility that blood flow in the region where is activated during performing the MA could be maintained to meet metabolic demand during MA even though the increase in MBV in MCA was attenuated after the exercise.

The mechanism(s) involved in the alteration of cerebral vascular response to mental task after the exercise is unknown. Cerebral vasculature is strongly related to arterial partial pressure of carbon dioxide (PaCO_2_). Although we did not assess the ventilation or PaCO_2_, MBV in the MCA at the pre-task baseline in the exercise trial did not differ from that in the control trial. This result does not imply that change in PaCO_2_ occurred 15 to 20 min after the exercise. Thus its effect on MBV in MCA is trivial, if any. Speaking, which was required to answer for mental arithmetic, could increase ventilation and consequently could decrease PaCO_2_ during MA. However, both trials applied the same task and thus the levels of PaCO_2_ should be similar between exercise and control trials. Thus, we can rule out the effect of decrease in PaCO_2_ induced by speaking. In turn, relatively greater magnitude of vasodilatation in some regions other than cerebral and splanchnic vasculatures could indirectly alter the cerebral vascular response to mental task after the exercise.

MBV and VCI in the SMA 15 min after exercise did not differ from the pre-treatment baseline. This was consistent with a previous study reporting that neither vasoconstriction nor vasodilation occurred in the splanchnic region during the post-exercise period [23]. On the other hand, MBV and VCI in the SMA in the control trial decreased from pre-treatment to the pre-task baseline, although there was no significant difference in pre-task values between exercise and control trials. Decreased MBV and VCI in the SMA in the control trial may be due to the non-significant but higher pre-treatment baseline value.

MBV in the SMA did not show significant change during mental task in both trials. The mental task performed in the exercise trial decreased VCI in the SMA in the same manner as in the control trial. Thus we cannot support our hypothesis that post-exercise attenuation of pressor response is associated with decreased vasoconstriction in splanchnic region, though there remains the possibility that vasoconstriction would have been attenuated at the same time if post-exercise attenuation of the pressor response occurred.

We did not record the diameter or calculate the blood flow in MCA and SMA; however, it was reported that the MCA diameter remains relatively constant during PaCO_2_ change [24] and that the SMA diameter did not change after meal ingestion [16]. In addition, vasoconstriction occurs mainly in resistance vessels, such as small arteries and arterioles which are innervated by the vasoconstrictive sympathetic nerve [25]. Since the target vessels are not resistance vessels, their diameter was unlikely to change during the present protocol. Thus, the flow velocities in both arteries are directly related to the flow volume.

## Conclusion

In conclusion, acute exercise adopted in the present study did not attenuate the pressor response and splanchnic vasoconstriction after exercise, but suppressed the blood flow increase in the cerebral vasculature during a subsequent mental task, implying that acute exercise can modify the cerebral vascular response to mental tasks. Further studies are needed to elucidate the mechanism(s) involving in the modification of cerebral vascular response to post-exercise mental tasks, and to reveal the responsible region(s) for the reduction of total peripheral resistance and/or attenuation of the pressor response during post-exercise mental tasks.

## Competing interests

The authors declare that they have no competing interests.

## Authors’ contributions

NS designed and coordinated the study, carried out the experiment, and drafted the manuscript. TI helped to measure the cerebral blood flow and to analyze the data. NH participated in the design of study and coordination and helped to draft the manuscript. All authors read and approved the final manuscript.
